# Topics of Public Interest

**Published:** 1904-06

**Authors:** 


### TOPICS OF PUBLIC INTEREST.

### Afterthoughts: A Correspondence.

By A. JACOBI, M. D.,
Chairman of the Joint Conference Committee.

(*Brooklyn Medical Journal*, May, 1904.)

"A LONG step in advance has just been taken by the agreement of the New York State Medical Association and the Medical Society of the State of New York to join hands and to form an organisation worthy of the twelve thousand physicians of the Empire State."—Frederick Holme Wiggin, president's address, *N. Y. State Journal of Medicine,* November, 1903.

"I feel that we should consider not only what is for our present interests, not our feelings for the present time, but should take into consideration the question of the future welfare of the medical profession of the State of New York."—William H. Thornton, presidential address, March 21, 1904.

EDITOR OF THE BROOKLYN JOURNAL:

*Dear Sir*—You have been good enough to address me lately in reference to the unification of the medical profession of the

State of New York. My first impression when I read your letter was that I had nothing to say in addition to the reports made by the committees of the Medical Society of the State of New York and of the New York State Medical Association. They have been published and appreciated, both for their historical value and the lessons they teach. Still there are several reasons why I should apply to you for a hearing in regard to the evolution which has taken place under our eyes and with the cordial support of most of us. Like those many who take a warm interest in the welfare and the progress of the profession—their number is increasing with the knowledge of its beneficial influence in shaping the sanitary, political and social destiny of the people—I have rejoiced in the success of the endeavors to consolidate our two large medical bodies so as to continue and reëstablish on its old firm foundation the Medical Society of the State of New York, and to fortify it by the democratic principles elaborated in the organisation of the American and the New York State Medical Associations. Some of our large journals,—the *Medical News,* the New York and Philadelphia *Medical Journal,* the *Journal of the American Medical Association, American Medicine* and others,—have made their readers fully acquainted from time to time with the labors of the two committees appointed by the Medical Society of the State and the New York State Association. They were not always light. It took time, patience and persistency to arrive at results which were to meet with unanimous acceptance.

What is this unanimity due to? To no single person or number of persons. History is not made by individuals, but is the visible result of a gradual evolution, the appreciation of which may be obscured or retarded by the personal predominance of an individual, but the latter is rather the creation of his time and surroundings, than the "time" the creature of an individual. The history of the medical profession and its progress are not the work of personal influences, but of the advancing opinion and demands of that very profession at large. In the State of New York we have experienced many such instances. The improvement in medical teaching and mainly the lengthening of college courses from two to three, and from three to four years are due to the pressure public medical opinion exerted on the schools. They were influenced by the demands of the practitioners as represented in the large societies. We never knew of an instance in which the profession gave up its fight for improvement; but those who have lived long enough remember quite well that once a medical faculty appeared before its class of students to announce that the term of study after having been lengthened would again be shortened, and the curriculum crippled. Then we remember the constant endeavor to establish state examinations, which must be passed previous to the granting of the license to practise medicine, also the increase of the requirements for matriculation. We also remember not only that some of the medical schools lent no friendly hand, but that the profession at large, represented in the Medical Society in the State of New York, had to fight the battle of medical progress against the schools. Many may even know that representatives of a few colleges labored hard to repeal a law which appeared to them to restrict their privileges and to curtail their advantages.

It is proper that medical men should recognise the fact that their welfare rested and rests on their own exertions, and not on any established and controlling power outside. It is the democratic spirit that rules the social and political existence of our people which has been governing the action of the medical masses. No American used to self-government and self-help waits either for outside pressure to enable him to overcome difficulties or to attain his legitimate ends, both in politics and in the interest of scientific or professional affairs. It is this democratic principle underlying the activity of our people that has been powerful in the development of the medical profession.

That explains what happened twenty-two years ago, and again this year. Both separation and consolidation were the doings of the medical men of the state. There were spokesmen, but no self-styled leader to initiate the several movements that have shaken the profession to its very foundation. Both parties were fired by what was to them a sacred principle. After nearly a generation we find ourselves on the same platform. The American Medical Association has given official recognition to the fact that cultured men must not be whipped into line by ironclad laws, but are expected to obey the rules of gentlemanly behavior on principle, and that ethics are not the outgrowth of fear but of heart and intellect. What is it to us that there are irreconcilables? A few morbid natures will tell you that you have been "snared" and "betrayed" when you feel like taking the hand of your brother from whom you are no longer estranged by principle or other interests. A few others are wise enough in their generation to bend before the storm of universal enthusiasm, and claim the credit of unification which they have opposed obstinately and bitterly.

After all, it was the medical profession which was unanimous as to the necessity of burying the hatchet which threatened to become a boomerang. Mainly to the young men is due the credit of insisting upon belonging to a united profession, not very vociferously but unmistakably. To them belongs the future, to them also the future leadership in the counsels of the profession of the state. Older men who have been through it all have reason to be satisfied with the share they took on either side, both in the battle for what they considered principle and in the reconciliation of the no longer divided parties, and are quite willing to let those work ahead who have both the strength and good will. I know I was correct when a few months ago I said to you all: "After unification we shall be satisfied that nothing will ever sever us again. For there will never be a diversity of principles like twenty-two years ago. There will be a common faith, aim, and altar. We shall take in the medical brotherhood the place which belongs to us, and work for the ideals—scientific, moral and practical—such as no other profession can realise, together with our peers all over the Union. We must or need split up into parties nevermore. It is only to the undivided and indivisible body medical that the American people will look for authoritative guidance in matters of sanitation, education and forensic legislation. What a Greek sage demanded should and may become true, viz.: "that the commonwealth will be advised and governed by the physician."

*Quod felix faustumque sit.* Very little is required, now that the Medical Society of the State of New York and the New York State Medical Association have unanimously adopted the reports and resolutions of the Joint Conference Committee, and the ratifications of the county societies and county associations arrive in rapid succession. An application to the Supreme Court, as the committee has promised, will be made early in May. It is a pleasure to acknowledge the services rendered by a Brooklynite for the purpose of facilitating that final step. Dr. James H. McCabe, one of your members of the Senate, volunteered to take charge and steer safely the following brief bills, which were considered desirable by many of us. They are as follows:

An Act to amend chapter three hundred and seventy-nine of the laws of eighteen hundred and eighty-five, entitled, "An Act regarding membership in the Medical Society of the State of New York."

*The People of the State of New York represented in Senate and Assembly do enact as follows:*

Section 1. Section one of chapter three hundred and seventy-nine of the laws of eighteen hundred and eighty-five, entitled "An act regarding membership in the Medical Society of the State of New York," is hereby amended so as to read as follows:

Section 1. The Medical Society of the State of New York shall have full power to elect such members as may be provided for by the constitution and by-laws of said Medical Society, said Medical Society being hereby empowered to fix and determine the qualifications and conditions of membership therein and to regulate and control its own membership.

Sec. 2. Section two of said act is hereby amended so as to read as follows:

Sec. 2. All acts and parts of acts, whether general or special, inconsistent with this act, are hereby repealed.

Sec. 3. This act shall take effect immediately.

An Act to amend chapter ninety-four of the laws of eighteen hundred and thirteen, entitled "An act to incorporate medical societies for the purpose of regulating the practice of physic and surgery in this state."

*The People of the State of New York represented in Senate and Assembly do enact as follows*:

Section 1. Section fourteen of chapter ninety-four of the laws of eighteen hundred and thirteen, entitled "An act to incorporate medical societies for the purpose of regulating the practice of physic and surgery in this state," is hereby amended so as to read as follows:

14. It shall be lawful for the Medical Society of the State of New York and the respective county medical societies to adopt constitutions and by-laws relative to the admission and expulsion of members and the regulation of their affairs; provided, that the constitutions and by-laws of county medical societies shall not be contrary to or inconsistent with the constitution and by-laws of the Medical Society of the State of New York, *except that each county medical society shall have full and unrestricted power of disposition and control over its real and personal property.*

Sec. 2. Section five and section seven of chapter ninety-four of the laws of eighteen hundred and thirteen, passed April tenth, eighteen hundred and thirteen, are hereby repealed.

Sec. 3. This act shall take effect immediately.

Both bills have passed the legislature, and are in the hands of the Governor for his signature. There seems to be no reasonable doubt but that they will be signed before the expiration of the time limit. They will complete the legislative measures which were required, or considered advisable, in the interest of the medical profession of the State of New York.

